# Special Issue: Prematurity, Preterm-Born Adults, and Long-Term Effects on Children and Adults

**DOI:** 10.3390/children10060989

**Published:** 2023-05-31

**Authors:** Vasantha H. S. Kumar

**Affiliations:** Division of Neonatology, Department of Pediatrics, John R. Oishei Children’s Hospital, University at Buffalo, 1001 5th Floor Main Street, Buffalo, NY 14203, USA; vkumar3@buffalo.edu; Tel.: +1-716-323-0260; Fax: +1-716-323-0294

The incidence of preterm births is increasing globally, with increasing survival into adulthood. Additionally, advances in neonatal care have led to improved survival of premature infants with significant morbidities. However, prematurity is a risk factor during development and may be related to adverse long-term outcomes. This Special Issue addresses the problems of preterm infants as they grow into children and adults.

It is exciting to present a volume of close to ten articles that address the issues of preterm infants as they grow into childhood, adolescence, and adulthood. Extremely premature infants are at particular risk for bronchopulmonary dysplasia (BPD). The definition of BPD has evolved since Northway et al. first described it 50 years ago. The benefit of BPD diagnosis is its ability to predict long-term respiratory and other health outcomes. However, with premature infants growing into adults, the variables affecting respiratory outcomes cannot be limited by the presence and absence of BPD. Jain et al. provide an overview of the long-term respiratory outcomes in infants with BPD and discuss the role of other modifiable or nonmodifiable factors affecting respiratory health in preterm infants [1]. Early life origins of adult-onset pulmonary diseases such as chronic obstructive pulmonary disease (COPD) with multiple prenatal, neonatal, and postnatal factors play a critical role in developing suboptimal pulmonary function and COPD in preterm-born adults [1]. Their study also emphasizes the urgent need to establish a pathophysiology and pulmonary function-based definition of BPD to help better predict long-term morbidities.

Reliable markers of BPD that predict the long-term consequences of respiratory and cardiovascular morbidities in preterm-born adults are evolving. For example, Henckel et al. compared YKL-40, a serum biomarker associated with airway remodeling and vascular smooth muscle proliferation, with relative telomere length in children at ten years of age with BPD [2]. This study is remarkable as it attempts to use altered lung development in BPD in predicting long-term outcomes. Research into biomarker development will help us understand the long-term relationships between lung development and its effect on lung function. 

Ideally lung function tests must be easy to perform, inexpensive and should have the ability for widespread implementation in all resource settings. Advances in technology should facilitate routine assessment of lung function to better understand airflow dynamics in infants and children born preterm. BPD and viral infections further complicate the development of airways and impact lung function. In a review by Gunawardana et al., poorer lung function, as assessed using pseudorandom noise (PRN)-forced oscillation technique (FOT), was not associated with a more significant burden of respiratory symptoms [3]. Nevertheless, there was insufficient evidence to determine whether PRN FOT performed better than other lung function tests [3]. This review article is a step in the right direction for further research into noninvasive and straightforward lung function testing in prematurely born children.

The growth and development of infants born premature is of utmost importance. Morbidities such as BPD, intraventricular hemorrhage (IVH), and periventricular leukomalacia (PVL) result in complications such as, feeding issues that impact growth and development. In this Special Issue, three preeminent articles address how preterm infants with short-term morbidities and neurodevelopmental problems affect the growth and development of these infants. Staub et al. compared blood pressure and kidney markers in adolescents born preterm with term control infants [4]. They found that male preterm boys had significantly higher systolic blood pressures than females, which was inversely correlated with birth weight and gestational age [4]. The finding that males tend to fare worse than females is interesting. However, the relationship between long-term outcomes and gender needs further research. Similarly, urinary kidney function biomarkers were higher in preterm infants, indicating long-term effects on kidney function from altered kidney development [4]. The results of altered kidney function on systemic blood pressure and cardiovascular morbidity need further exploration.

Kamity et al. conducted a detailed analysis of feeding issues in preterm infants and a systematic approach to evaluating and managing feeding issues in preterm infants [5]. Oral feeding problems are underappreciated, and the challenge of feeding the preterm infant needs more focused research. Early diagnosis and management of feeding issues will help in better overall growth and development of organ systems. Their review is an excellent synopsis of feeding issues in preterm infants and, hopefully, encourages more research into feeding-related problems in premature neonates. More such studies

The protective effects of human milk are well known, and exclusive use of mother’s own milk and donor human milk is becoming more widespread. Despite being expensive, human milk-based fortifications such as Prolacta are used in the NICUs to decrease the incidence of necrotizing enterocolitis (NEC) and its effects on long-term outcomes. The positive impact of human milk on long-term effects on organ systems, especially in premature infants, is an area of active research. With the ease of availability of human milk and enormous benefits, its use should be encouraged, particularly in rural and resource-poor settings. Chen et al. revealed that milk banks may facilitate the growth of very preterm infants in resource-poor environments [6]. The availability of human or donor milk through milk banks will go a long way in promoting growth and development and decreasing morbidities such as gastrointestinal and respiratory illness in children in developing countries.

Two studies with their focus on the eye are of particular interest. With an extensive focus on the retinopathy of prematurity, less research has been focused on other related eye problems, especially in children and adults born preterm. Corneal thickness alterations at 40 to 80 years in low-birth-weight infants are fascinating for their longevity of follow-up [7]. Longitudinal studies looking at problems of organ and organ systems over life span will help to better understand aging of former preterm infants. Similarly, Achim et al. revealed earlier anterior chamber angle width closure in adults born preterm with advanced stages of ROP [8], indicating that early detection and close follow-up are essential in these infants.

Close attention to neurodevelopmental issues in the routine follow-up of premature infants is essential for early intervention. For example, Helin et al. assessed motor performance, perceived loneliness, and social competence in mid-childhood [9]. Recognizing motor and neurodevelopmental issues is essential to provide interventions and support to prevent social exclusion.

I am honored to be the Guest Editor for this Special Issue dedicated to adults born preterm. The articles and research presented in this collection aim to advance the care of preterm infants. I congratulate the authors and their team for contributing to this Special Issue in *Children*, as the inspiring topics covered in this issue highlight the ongoing interest in promoting the growth and development of premature infants as they grow into children and adults. Further research into quality-of-life issues and promoting healthy lifestyles would help premature infants to grow as productive citizens of the society. 
